# How Do Patients and Health Workers Interact around Malaria Rapid Diagnostic Testing, and How Are the Tests Experienced by Patients in Practice? A Qualitative Study in Western Uganda

**DOI:** 10.1371/journal.pone.0159525

**Published:** 2016-08-05

**Authors:** Robin Altaras, Anthony Nuwa, Bosco Agaba, Elizabeth Streat, James K. Tibenderana, Sandrine Martin, Clare E. Strachan

**Affiliations:** 1 Malaria Consortium, Kampala, Uganda; 2 National Malaria Control Programme, Ministry of Health, Kampala, Uganda; 3 London School of Hygiene and Tropical Medicine, London, United Kingdom; Academic Medical Centre, NETHERLANDS

## Abstract

**Background:**

Successful scale-up in the use of malaria rapid diagnostic tests (RDTs) requires that patients accept testing and treatment based on RDT results and that healthcare providers treat according to test results. Patient-provider communication is a key component of quality care, and leads to improved patient satisfaction, higher adherence to treatment and better health outcomes. Voiced or perceived patient expectations are also known to influence treatment decision-making among healthcare providers. While there has been a growth in literature on provider practices around rapid testing for malaria, there has been little analysis of inter-personal communication around the testing process. We investigated how healthcare providers and patients interact and engage throughout the diagnostic and treatment process, and how the testing service is experienced by patients in practice.

**Methods:**

This research was conducted alongside a larger study which explored determinants of provider treatment decision-making following negative RDT results in a rural district (Kibaale) in mid-western Uganda, ten months after RDT introduction. Fifty-five patients presenting with fever were observed during routine outpatient visits at 12 low-level public health facilities. Observation captured communication practices relating to test purpose, results, diagnosis and treatment. All observed patients or caregivers were immediately followed up with in-depth interview. Analysis followed the ‘framework’ approach. A summative approach was also used to analyse observation data.

**Results:**

Providers failed to consistently communicate the reasons for carrying out the test, and particularly to RDT-negative patients, a diagnostic outcome or the meaning of test results, also leading to confusion over what the test can detect. Patients appeared to value testing, but were frustrated by the lack of communication on outcomes. RDT-negative patients were dissatisfied by the absence of information on an alternative diagnosis and expressed uncertainty around adequacy of proposed treatment.

**Conclusions:**

Poor provider communication practices around the testing process, as well as limited inter-personal exchange between providers and patients, impacted on patients’ perceptions of their proposed treatment. Patients have a right to health information and may be more likely to accept and adhere to treatment when they understand their diagnosis and treatment rationale in relation to their perceived health needs and visit expectations.

## Introduction

The presumptive, empirical treatment of malaria was common place in countries across Africa until relatively recently. In 2010, the World Health Organization (WHO) changed policy guidance, recommending parasite-based confirmation of diagnosis in all patients suspected of having malaria prior to treating [1, 2]. Since 2012, this recommendation has been promoted through WHO’s ‘test, treat and track’ strategy [3]. Routine adoption of parasite-based diagnosis is important so as to improve quality of care, avoid over-diagnosis of malaria cases which can lead to inappropriate or delayed treatment, and reduce wastage of anti-malarial drugs [4]. In recent years, most African countries with a continued malaria burden have adopted policies of parasitological diagnosis and implemented programmes aimed at expanding access to and use of diagnostic testing in both the public and private sectors. This has in large part been achieved through the extensive deployment of malaria rapid diagnostic tests (RDTs) which can be effectively performed even at lower levels of the health system [5, 6].

The routine use of RDTs in the management of patients with fever has represented a new approach in contexts with minimal exposure to diagnostic technologies. In settings such as rural Uganda, where clinicians at low level health facilities have long been accustomed to dispensing drugs in accordance with patient complaint [7], the use of a diagnostic test introduces significant changes in care practice. In areas where malaria has long been the most common outpatient diagnosis, confirmatory testing often alters the diagnostic outcome, with many suspected cases testing negative [5], frequently contradicting both patient and healthcare provider expectations. Patients are likely to require additional information from providers as part of routine practice, relating to the testing purpose, process, actual results and interpretation of the meaning of results in relation to the diagnostic outcome.

Communication is an important component of patient care; quality of care can be defined as a framework comprising the structure of health care, the process of actual care given and received, and the outcomes of the interaction between individuals and a health care system [8–10]. The effectiveness of inter-personal care is suggested to be as important as the effectiveness of clinical care when evaluating quality of care received [11]. Effective patient-provider communication leads to improved patient satisfaction, which is recognised as a key contributing factor towards better health outcomes through its effect on increasing access to care, raising demand for services, and improving treatment adherence [12–16]. How healthcare providers prepare patients for testing, explain the rationale and implications of testing, respond to patients’ questions or concerns, and communicate about test outcomes and subsequent treatment may all be important for influencing patient perceptions of, and satisfaction with, the diagnostic process.

The evidence further suggests that interactions with peers and patients, and voiced or perceived patient expectations, can influence provider treatment decision-making across a variety of contexts [2, 17–21]. Perceptions of patient demand for treatment, coupled with perceived patient rejection of test results, for example, may sometimes drive providers to prescribe anti-malarials to patients who test negative [2]. Understanding patient perceptions of testing and treatment is therefore important for promoting the successful scale up of the universal test and treat strategy.

While the last five years has seen a growth in literature exploring healthcare provider practices around rapid testing for malaria [2, 17, 18, 20, 22–27], how providers and patients *interact and engage* around RDT use has received less attention. To date, assessment of patient perceptions regarding the introduction of RDTs has largely focused on acceptability and trust of test results [19, 26–31], with positive reactions to RDTs and high levels of patient acceptability generally reported, particularly where testing has been established beyond an initial trial period. However, all consultations are a complex interaction between patient and clinician [20] and it is important to understand not only whether tests are acceptable or not to those receiving them, but also how the tests, the testing process and the surrounding patient-provider encounter and exchange are experienced by patients in practice and how this may affect patient perceptions of treatment (and subsequent adherence).

A qualitative study, incorporating in-depth interviews of patients/caregivers and observation, was conducted to explore how healthcare providers and patients interact around rapid diagnostic testing during clinic visits for fever, and how patients perceive and interpret the testing and treatment process. The study was conducted as part of a larger study which explored the factors driving providers to prescribe anti-malarials to patients with negative RDT results [2]. As far as the authors are aware, there has been no other qualitative observation conducted in this area. The study highlights the urgent need for providers to adopt a more dialogical and patient-centred communication approach, supported by clear protocols and communication aids, which clarifies the diagnostic and treatment process and addresses patients’ information needs. Given RDTs are the first point-of-care diagnostic to be introduced at many lower level health facilities, experience in their use may also have broader implications for communication practices around the introduction of other technologies or diagnostics in settings with limited resources.

## Materials and Methods

### Study setting

The study was carried out in Kibaale district, a remote, rural area in mid-western Uganda. The estimated population of 613,200 is predominantly located in dispersed villages and engaged in subsistence agriculture. In Uganda, all primary healthcare services (including malaria diagnosis and treatment) are provided free-of-charge at public health facilities. Artemisinin-based combination therapy (ACT) was introduced as treatment for malaria in 2006, with artemether-lumefantrine (AL) (Coartem^®^) as the first-line treatment for uncomplicated malaria [32]. In 2011, a histidine-rich protein 2 based RDT (SD BIOLINE malaria Ag-Pf, SD 05FK60), was introduced into 30 public health facilities which lacked functional microscopy, ahead of a planned nationwide roll out. (The process for introducing RDTs in the district has been described elsewhere [2].) At a similar time, RDTs were also introduced as part of an integrated community case management (ICCM) programme with community health workers, known in Uganda as Village Health Teams (VHTs), which contributed to raising community awareness of testing.

### Sampling

This study drew on the same sampling approach and patient sample as the main study [2]. Using prescription data from outpatient registers over a two month period, lower level health facilities were purposively sampled according to ‘prescribing performance’ (proportion of RDT-negative patients prescribed anti-malarials), with the aim of exploring a range of clinician prescribing behaviours [2]. This resulted in a selection of 12 facilities, including seven Health Centre (HC) IIs and five HC IIIs. All prescribing clinicians at the 12 health facilities were targeted for observation, with the aim of observing three fever cases per provider. Patients were selected for observation on a rolling basis at the point of care. All observed patients (or caretakers of child patients) were targeted for semi-structured interview.

### Observation and patient/caregiver interviews

The main study reported findings from observation (related to case management) and healthcare provider interviews; this paper reports findings from observation (related to communication) and patient/caregiver interviews. Data collection was carried out in November and December 2011, approximately ten months following the introduction of RDTs, a cross-sectional point when their use was expected to have been integrated into routine practice and there would be some awareness and expectation of routine testing for malaria among patients presenting with fever [2]. Observation and semi-structured interview guides were pre-tested at health centres in two neighbouring districts, as under the main study. The data was collected by four social scientist research assistants with experience in qualitative research and a range of language skills relevant to the setting. All research assistants received two days training on the specific technical scope of the study and use of the data collection tools.

Observation was conducted by two of the four research assistants. During the preparatory phase, the two observers simultaneously observed three cases and then systematically compared transcripts for discrepancies, with the aim of reducing inter-observer variability [2]. The observation guide sought to capture exchanges and interaction between the healthcare provider and the patient throughout the care process. Provider-recorded diagnosis or patient complaint, RDT result, and prescription information were also abstracted from patients’ record books. Observation procedures have been described in detail in relation to the main study [2].

Patient interviews were audio-recorded and conducted in the language of the respondent. The main languages spoken in the area are Runyoro-Rutooro, Rukiga, Rufumbira, Runyankole, Rukonjo, and Runyarwanda-Kinyarwanda, though Luganda and Kiswahili are also used. Given the range of languages, research assistants were trained to conduct oral translation of the patient interview guide at the point of interview. Once an observation was completed, the observed patient (or caregiver) was invited for interview with the observer, accompanied by a second research assistant. For observed child patients, the interview was conducted with the accompanying caregiver. Scope of enquiry included demographic characteristics, reason and expectations for visit, choice of health facility and prior care-seeking experience there, familiarity and prior experience with RDTs, and present testing and treatment experience (communication of test purpose, perceptions of testing, experience with receiving and understanding test results, perceptions of test results, understanding and acceptance of treatment provided, and overall satisfaction with visit).

Observation notes and verbatim interview transcripts were prepared in English in the field throughout data collection. To avoid loss of meaning and interpretation bias, key terms in local languages were retained alongside the English translations. Sections of the transcripts were compared with audio-recordings for review of translation accuracy.

### Analysis

Analysis followed the ‘framework’ approach [33], whereby a pre-existing coding frame was developed based on the scope of enquiry to which codes were added on review of the data. All data were coded and indexed in Excel (Microsoft) and analysed according to the most salient themes by RA (MPH), with regular review and discussion with CS (MPH) and AN (MD, MPH). Content analysis of observation data also used a summative approach in order to assess relative frequency of certain aspects of communication (e.g. linguistic phrases and keywords used to communicate the purpose of testing, RDT result, diagnosis or treatment plan). Summative findings were then interpreted contextually. Triangulation of individual-level data (observed interaction and linked patient/caregiver report) was also performed for validation and deeper exploration of key themes.

### Ethics, consent and permissions

Ethical approval was granted by the Uganda National Council for Science and Technology (UNCST, HS 1009). Informed oral consent was obtained from all healthcare providers prior to observation. To avoid altering routine clinical practice, clinicians were provided with consent statements and asked to inform and consent patients to the presence of the observer during consultation. If any patient had not consented to the presence of the observer at any time, the observer would have simply left the room and returned at the start of the next consultation, though this situation did not arise. Oral consent for interview, audio-recording and record book review for both RDT result and prescription information was subsequently obtained from the patient or caregiver. In this case, oral consent was requested for reasons of comfort given the low literacy among the general patient population and associated challenges with reading and signing a consent form. Participant consent (from both providers and patients/ caregivers) was witnessed by the note taker and documented in the interview notes: a series of tick marks were made as each point of consent was explained and the final consent for interview or observation noted. Consent statements also included information on the study’s broad aims, confidentiality, respondent rights and uses of the data. Unaccompanied minors were not observed.

## Results

This section describes the observed communication practices between the healthcare provider and patient throughout the care process and reports on the findings from the patient/caregiver interviews, giving insight into how patients perceived and interpreted the testing and treatment process.

### Study participants

As reported in the findings from the main study, a total of 55 patients were observed across 22 healthcare providers [2]. No observed patients or caregivers refused to be interviewed. All patients were tested for malaria with an RDT with slightly more than two-thirds (n = 38) testing negative. Key demographic characteristics of participating patients are provided in Table 1; characteristics of the observed clinicians have been described elsewhere [2].

**Table 1:** Demographic characteristics of observed patients.

	Number of patients (%)					
	RDT-negative		RDT-positive		Total	
	N	%	n	%	N	%
**Female**	20	57%	15	43%	35	64%
**Male**	18	90%	2	10%	20	36%
**Adult**	27	82%	6	18%	33	60%
**Child**	11	50%	11	50%	22	40%
**Total**	38		17		55	

### Overall care process

In all of the observed health facilities, RDTs were systematically performed on patients complaining of fever. Generally, the RDT was performed after patient registration and/or consultation. There were three providers, however, who employed the RDT as a *de facto* screening test, rather than a confirmatory diagnostic. At most of the observed health facilities (10), RDTs were performed in the same room as patient registration and consultation. However, the RDT was performed by a different health worker from the consulting provider in nearly half of observed cases. In these cases, patients were either sent to a testing space within the same room or told to leave the room and wait to be called back in for testing. At health facilities with only one provider, some opted to ‘batch’ fever cases requiring testing and others tested patients during the consultation. Following testing, almost all patients and caregivers were told to “*go out and wait to be called*” or were asked to wait in the same room where they were tested (often remaining in the consultation room as health workers proceeded to register and/or consult other patients). Frequently, several activities were occurring at once in the same room, making the space congested and effectively eliminating the possibility of a confidential patient-provider exchange.

One of the health workers was on the clerking table, another one on RDTs and transferring data to the outpatient register, while the third one was dispensing the drugs to the patients. Everything is done from the same room (consultation, RDT, prescription and dispensing of drugs). The table used for clerking (registration) was also used for dispensing the drugs and the RDTs were being done on the examination bed just behind the clerking table. There is no privacy in this health facility because there are three health workers all seated in the same room, with each attending to a patient(s) and also the other patients are seated very close to the table where consultation is done. At one point, there were eight people in the room.

*[Observation notes*, *HCII]*.

On average, patients encountered 1.9 health workers during the course of their visit; this was the same at both HCIIs and HCIIIs, although a greater proportion of observed patients at HCIIIs saw two or more health workers (70% vs. 55%). Many patients did not re-see the health worker who conducted their initial consultation and test. In some facilities, task shifting was also common during the course of the day, with the health worker who conducted patient registration in the morning subsequently shifting to pharmaceutical dispensing or recording patient outcomes in the outpatient register later in the day. Although the average length of patient visit (time from beginning of consultation to treatment dispensing) was 1 hour 17 minutes, the duration of the clinical encounter (time spent directly interacting with a health worker for registration, consultation, testing and dispensing) ranged from three to 35 minutes [2].

### Talking about fever and malaria

Patient-provider communication surrounding malaria testing was complicated by linguistic issues. In many of the languages spoken in the study area, the concepts of fever and malaria are frequently expressed using the same word, *“omuswijja”*. Although “*omuswijja”* is a non-specific term which may refer to any febrile illness, it may also comprise other symptoms that overlap with the clinical presentation of malaria [34]. Malaria can be more precisely expressed as “fever/malaria caused by mosquitoes” (*“omuswijja gw’emibu”*, taking one example in Runyoro-Rutooro). However, few providers or patients seemed to make this distinction, at times employing the word “*omuswijja”* with the intent of conveying malaria, and at other times, overlooking any difference in meaning.

### Patient agendas and acceptance of testing

When asked about expectations for their visit, patients and caregivers mostly talked about receiving medicine to treat their problem or illness as identified by the health worker. About half of all patients specifically mentioned expecting to receive “*drugs for fever/malaria*” or specific drugs. Just a few patients mentioned an expectation of being tested.

The majority of patients had had some prior exposure to RDTs, through having been tested themselves, having had a family member tested, or having seen the test performed by VHTs in their village. Just three of the 55 patients reported no experience with or awareness of RDTs prior to their visit. There was wide reporting of the principle of “*testing before treating*”, with reference to messages from radio shows, health workers or key figures in the community.

“I have never had this test done. I always bring my children to test but not myself. I have heard about this test being advertised on radio, even the VHTs in the village carry out these tests among children below five years. Even health workers encourage people to test first before getting medicine…The [radio] programme was about the importance of testing before taking drugs and they also said that it is a standard procedure to test before getting malaria tablets.”

*[PT01*, *RDT-negative male adult*, *seen by HW01*, *nurse in charge*, *HCIII]*

Patient acceptance of testing appeared to be high. Most patients appeared to welcome the test in order to know the illness underlying their symptoms and to enable receipt of appropriate treatment, rather than medication based on “*guessing*”.

“I felt happy because I knew that I would be given the right treatment basing on what the results would show…Before the introduction of this test, we were given drugs without testing and most people would either take a long time to improve or they would totally fail to heal. But now these tests guide health workers in administering the right drugs hence making their work easier.”

*[PT08*, *RDT-positive female adult*, *seen by HW03*, *nursing assistant*, *HCIII]*

### Test purpose

Both observation and patient interviews highlighted little explanation by the healthcare provider about the purpose or rationale for testing. Prior to testing, less than half of all observed patients were told something about the test purpose that specifically related to seeking confirmation of malaria infection. Healthcare providers more commonly mentioned non-specific aims such as “*testing*” or “*checking blood*”. When malaria was mentioned, a non-specific term for *“fever*” was often used interchangeably with “*malaria*”. In two cases, it was suggested by providers that the test could potentially enable diagnosis of multiple infections (“*find the cause of the sickness*”). In nine cases (across seven different providers), patients received no communication prior to testing; the health worker simply initiated a blood draw without speaking to the patient. For some patients, the lack of communication seemed of little consequence given they already felt familiar with the testing process, but others reported that more information on the reason for testing would have been useful.

“I don’t think the health worker explained to me clearly what the test was for. He just told me that the child was going to be tested for malaria caused by mosquitoes. I would like him to explain what would happen if the result was positive or if it was negative and which treatment he would give him.”

*[PT21*, *Caregiver of RDT-negative male child*, *seen by HW07*, *nursing assistant*, *HCIII]*

Vague or limited explanations of test purpose appeared to contribute to confusion over what the test was able to detect, with many patients suggesting it could identify all febrile illnesses, differentiate between types of malaria/fever, or identify any disease. Two patients believed they were being tested for HIV and two further patients reported that the provider mentioned differentiating malaria and typhoid. A clear overlap between patients reporting insufficient explanations of test purpose and those who were unclear on the purpose of testing was observed.

“The health worker told me that if am complaining of malaria, I would be tested first to determine the type of malaria and I understood that I would get the right treatment for the type of malaria that I have.”

[PT55, RDT-negative female adult, seen by HW22, nursing assistant in charge, HCII]

“I did not know they were testing for what. I thought they were testing for HIV or what. This is the first time to hear that this thing [points at the RDT in the interviewer’s hand] tests for malaria. They tested me without telling me anything, wrote in the book and gave me medicine.”

*[PT42*, *RDT-positive female adult*, *seen by HW18*, *nursing assistant*, *HCII]*

Few patients were observed to, or reported, asking questions at the time of testing. Most patients reported they had no questions to ask, given their familiarity with the testing process, though some expressed reticence to ask questions, mostly due to a lack of opportunity or comfortable environment. Providers often used an instructional tone when introducing the testing process (e.g. *“Wait here to get tested”*); this emphasised authority may have discouraged patients from asking questions.

“I am going to check your blood for fever (omuswijja).”

[HW19, nursing assistant acting in charge, speaking to PT46, RDT-negative female adult, HCII]

“I had some questions but I didn’t ask because there were many people around and I thought they might hear what I had to say and since the health worker was alone working on all patients, I thought she might not have enough time to explain all that I wanted to ask. So I decided to keep quiet and reserve my questions for another visit.”

*[PT53*, *RDT-negative adult*, *seen by HW22*, *nursing assistant in charge*, *HCII]*

### Communication of test result

The majority of patients (around three-quarters) were either explicitly informed of their test result or were told that they did or did not have malaria or fever. Half of these communications were specific about the test: the provider reported a negative or positive test result or mentioned something related to “testing” when informing the patient that he did or not have malaria or fever. Among RDT-positive patients, the specific test result was mentioned in slightly less than half of encounters; however, all but four of the 17 patients who tested positive were told that they had malaria. The specific test result tended to be communicated more frequently to patients who tested negative: more than two-thirds of the 38 RDT-negative cases were informed of the result or an outcome associated with testing.

[No specific mention of test results, but confirmation of malaria diagnosis:]

“Your child has malaria and she is anaemic so am referring you to [name of] hospital.”

*[HW02*, *nurse-midwife at HCIII*, *speaking to caregiver of PT06*, *RDT-positive child]*

[Test result explicitly associated with outcome:]

“Blood tests are out, and they indicate that the child has fever caused by mosquitoes, so we are going to give you some medicines to give this child.”

[HW11, nurse at HCIII, speaking to caregiver of PT27, RDT-positive child]

“RDT results are out and they are showing that the child is negative and the child does not have fever. Now we are going to treat cough and give you medicine.”

*[HW14*, *nurse at HCIII*, *speaking to caregiver of PT33*, *RDT-negative child]*

The use of non-specific illness classification terms seemed to contribute to misunderstandings about the meaning of the test result.

The health worker informs the patient that he does not have “malaria” and the patient refuses the results saying, “What?”

H: “I have told you that the test results show that you do not have malaria fever.”

P (angrily): “But I have fever.”

H: “No you don’t have fever. So we have these tablets here for cough since it was the one which came first.”

P: “For you the only tablets you give are for malaria, what if I have other types of fever?”

H: “Here we do not test for typhoid fever and if you think you have typhoid you have to go and seek treatment from elsewhere.”

*[HW17*, *nursing assistant*, *HCII*, *and PT40*, *RDT-negative male adult]*

Gaps in communication of test results occurred across health facilities and providers: the 13 patients who were not informed of test results were distributed across eight health facilities and 12 providers, suggesting broad inconsistencies in routine practice. In some cases, absence of communication about results appeared to be related to organisation of service delivery and clinician workload. At health facilities where multiple health workers attended patients, gaps in responsibility with regards to communication were apparent, with documentation in the patient’s record book facilitating communication between health workers, perhaps obscuring the absence of verbal communication to the patient. In some cases, test results were communicated by the dispensing health worker, and in other cases, patients never learned their results.

Patient is called in the room at 13:05 and she enters and stands next to the health worker. The health worker does not tell her the results of the test but instead asks for her book: “Give me your book?”

*[Observation of PT51*, *RDT-negative female adult*, *seen by HW21*, *nurse in charge*, *HCII]*

Some patients reported reaching their own conclusions about the test outcome, based on the treatment received and their prior knowledge and experience with testing and taking anti-malarials, or from reviewing what had been recorded in their record book. However, in one case, a lack of direct communication on the test outcome led to complete misinterpretation by the patient; the patient was observed to have tested negative, but he believed that he had actually tested positive and had malaria. As with the initial testing process, patients rarely raised concerns or sought clarification on the test outcome.

“[Frowning]. I knew the child had malaria from the drugs that were given to me but the health worker did not tell me anything. Whenever I am given these drugs [she looks at the AL] after the child has been tested I just know the child has malaria. But that health worker did not tell me anything.”

[PT30, caregiver of RDT-positive female child, seen by HW13, nurse in charge, HCIII]

“Can I argue with the health workers who are trained? I don’t trust the results but I cannot challenge health workers who are trained.”

*[PT16*, *RDT-negative female adult*, *seen by HW06*, *nurse*, *HCIII]*

### Diagnosis—told what don’t have, but not what do have

Although more than two-thirds of patients who tested negative were informed of the test result, they were not always provided with an alternative diagnosis or an explanation of the rationale for treatment. Among the 38 patients who tested negative, clinicians were observed to provide three levels of diagnostic information:

1. Half (19) were told they did not have malaria and provided with a minimal or unspecific explanation about another possible cause of illness. In some cases, the provider just noted that he would therefore treat other symptoms or patient complaints.

“We are going to give medicine for fever and cough, so do not get worried, so you can now follow me so that I give you drugs.”

*[HW09*, *nursing assistant*, *HCII speaking to PT23*, *RDT-negative male adult]*

One patient found out what he was being treated for only after asking:

P: “So the tablets you have given me are for what?”

H: “For cough and flu.”

*[PT40*, *RDT-negative male adult and HW17*, *nursing assistant*, *HCII]*

2. More than a third (14) of patients who tested negative were told they did not have malaria, but were not given any alternative explanation for their illness. In many of these cases, drugs were simply dispensed.

“Now the RDT results are out and you do not have fever caused by malaria, so take this medicine [chlorphenamine], one tablet three times a day. Then this [paracetamol], two tablets three times a day, then this [trimethoprim-sulfamethoxazole], two tablets two times a day.” [Provider does not explain the different names of the drugs to the patient.]

[HW05, nursing assistant, HCIII speaking to PT15, RDT-negative female adult]

3. The remaining patients (5) were not provided with any diagnostic information.

Among patients who tested negative, a main point of frustration was the provider’s failure to reconcile the negative result with the patient’s symptoms: patients expected to be told more about their illness and expected the provider to explain why they had symptoms of malaria yet the results were negative. Some of these patients explained that they understood their symptoms could be caused by “*another disease*” or “*something else*” and that they were being given medicine for other symptoms or complaints, but remained dissatisfied at not being told more about their own diagnostic outcome.

“I was told that the results indicate that [name of child] doesn’t have malaria. I didn’t understand the results very well because my son develops high temperature especially in the evening and he also lost his appetite. So if the health worker says that he doesn’t have malaria, then I get confused and I don’t know what he is suffering from. The health worker told me that it could be the cough that is causing the high temperature and she added that she would give me medicine that will cure him. I needed to know what could be the cause of loss of appetite but I didn’t ask the health worker because there were many patients waiting to be treated.”

[PT21, caregiver of RDT-negative male child, seen by HW07, nursing assistant, HCIII]

“The nurse did not explain. She just told me that the child does not have malaria. That information was not enough, the nurse told me that the child does not have malaria and yet he convulsed last night and even has a high temperature now. What is the cause of that?”

*[PT18*, *caregiver of RDT-negative male child*, *seen by HW06*, *nurse*, *HCIII]*

### Trusting the test result

Overall, patients reported fairly high levels of trust in the test results, particularly among patients who tested positive, who commonly observed that the results “*confirmed expectations*”. Across all patients, factors that appeared to be important in fostering trust of results included prior positive personal or peer experience with testing and treating (“*other people come and get better”)* and a belief in technology (“*tests don’t lie”)*. Among those who tested negative, visualisation of the test results (seeing the lines on the cassette, observing other sick-looking patients who also tested negative), trusting health worker competency, and receiving a satisfactory alternative explanation for the cause of fever also appeared important in fostering patient trust in the result.

Generally, doubts about test accuracy were confined to patients who tested negative. Slightly less than a third of patients testing negative expressed doubts; this was particularly apparent among those who demonstrated confusion over what the test can detect and who felt a disconnect between feeling symptoms of malaria and not receiving an alternative diagnosis.

“I don’t know whether to trust the test or not because there are times especially in the evening when I feel feverish and get signs of malaria but if the test says that I am negative, then I don’t know whether to believe it or not.”

*[PT26*, *RDT-negative female adult*, *seen by HW10*, *nurse in charge*, *HCIII]*

### Benefit of testing

All patients who tested positive reported that there was some benefit to testing. Most patients who tested negative also perceived some benefits from testing, such as enabling the provider to “*treat what he knows”* or “*treat the right illness*”, as well as learning that they did not have malaria. However, some RDT-negative patients reported no benefit to testing, linking this closely to the absence of an alternative diagnosis or the receipt of treatment; a few patients indicated that there would have been a benefit to testing if they had tested positive.

“I didn’t benefit anything because I have been given medicine without knowing what I am exactly suffering from.”

*[PT51*, *RDT-negative female adult*, *seen by HW21*, *nurse in charge*, *HCII]*

### Treatment plan

Although many patients were not informed of the rationale for treatment, the dispensing clinician generally provided some explanation of how to administer prescribed medicines. However, healthcare providers rarely mentioned actions to be taken in case the patient did not improve. Referral was made in four of the 55 observed encounters, however there were no discussions relating to the execution of referral (which patients don’t always have the means to do).

Most patients expressed confidence that they would improve with the treatment received or that they were uncertain and would have to wait and see. Confidence in the treatment plan appeared to be fostered by belief in the ‘testing before treatment’ approach, a familiarity with and belief in the effectiveness of the medication received, and previous health recoveries following visits to the same health centre.

Patient dissatisfaction with treatment appeared to result primarily from the absence of an alternative diagnosis and perceptions of not receiving adequate treatment. These perceptions were influenced by patient expectations (desired treatment or expectation of receiving anti-malarials), patient understanding of treatment purpose, the adequacy (quantity and type) of drugs prescribed, and the availability of prescribed drugs at the health facility. While some patients expressed dissatisfaction over not receiving drugs, in part this appeared to reflect poor comprehension of the treatment plan, rather than not receiving AL or an alternative treatment. For example, one adult patient *[PT54*, *RDT-negative female adult seen by HW22*, *nursing assistant in charge*, *HCII]*, who was told that she did not have malaria but was not informed of a diagnosis or the purpose of prescribed medicines, expressed her frustration despite receiving trimethoprim-sulfamethoxazole, mebendazole, paracetamol and chlorphenamine (during interview, she was able to identify all of the drugs except trimethoprim-sulfamethoxazole). Similarly, two caretakers of RDT-positive children also expressed concerns about the treatment plan: one because she was not informed of the test result and the second because she did not receive all prescribed drugs and was not informed about the purpose of the drugs.

“I don’t think I received the right treatment because [name of child patient] was not given drugs. I was told to buy three types of drugs and am not even sure that [child’s name] will improve because she was not given anti-malaria drugs yet she has a high temperature and she is generally weak. I think it is not the right treatment because she was not given [AL] yet they have it in stock.”

[PT48, caregiver of RDT-negative female child, seen by HW20, nurse in charge, HCII]

“When they test you and find you with malaria, you are given drugs but when they test you and tell you that you do not have malaria, you go away without drugs. The health worker did not explain the results for me to understand so as not to go away with doubt.”

*[PT20*, *caregiver of RDT-negative male child*, *seen by HW07*, *nursing assistant*, *HCIII]*

In spite of reported doubts about proposed treatment plans, few patients were observed or reported raising questions or requesting alternative treatments. Some patients implicated themselves as passive recipients of care, appearing either intimidated by, or respectful of, the knowledge or authority of healthcare providers. Just one patient reported explicitly asking for AL after being informed that his results were negative.

“I don’t know whether I received the right treatment because the health worker tells me that the child will get better yet she has not given me drugs for malaria. So I am not sure the child will get better. Maybe after improving, I will know that the treatment was right…I didn’t ask for any other drugs because I feared the health workers since all of them were in the room. Maybe if I was alone with one health worker in the room, then I would ask for other drugs.”

[PT36, caregiver of RDT-negative male child, seen by HW15, nurse in charge, HCII]

“I don’t know whether I got the right treatment. I will only know that it was the right treatment after I totally cure. I asked for [AL] because it’s the only anti-malaria drug that doesn’t bring dizziness and body weakness but the health worker told me that she can’t give [AL] because the results indicate that I don’t have malaria.”

*[PT28*, *RDT-negative female adult*, *seen by HW12*, *nursing assistant*, *HCIII]*

## Discussion

Patient acceptance of testing appeared to be high, with most patients welcoming the idea of receiving treatment based on a confirmed diagnosis. This is congruent with findings across a range of contexts, including three earlier studies in Uganda (conducted formatively, shortly after RDT introduction and after one year of implementation), which all found that diagnostic testing was perceived as useful for reducing uncertainty around the diagnosis and so enabling appropriate treatment [28, 31, 35].

However, little, vague or no explanation of test purpose was found to be common among healthcare providers during consultations. This led to patients being misinformed over what the test can detect, such as different types of malaria or other, even multiple, infections. Conceptualisations of the RDT as a generic test able to identify *any* cause of illness, and not just malaria, have been reported elsewhere [20]. The use of non-specific illness classification terms also seemed to contribute to misunderstandings about the purpose of testing; confusion around, or amplified expectations of, what the test can identify could in time compromise acceptability given patient understandings may not match what the test can actually detect [20].

In a quarter of observed clinical encounters, patients were not directly informed of their test result or told whether or not they had malaria by a healthcare provider. Patients testing negative for malaria were commonly not given an alternative diagnosis, which frequently contributed to ongoing confusion about the cause of the symptoms which presented similarly to malaria. This lack of communication and explanation may contribute to a limited understanding of the meaning of the test result [20] and leaves room for patient misinterpretation (patients may believe the test reveals more than it does).

Communication of the outcome of the test was also often done without explicit mention of the test, particularly in the case of a positive malaria result. Using this opportunity to associate the test process with the test result may help to clarify the rationale for treatment, ultimately important in garnering acceptance in, and adherence to, the treatment plan. The link between the level of information received by the patient and the trust generated in relation to the proposed treatment has been discussed elsewhere; while talk itself can be therapeutic (e.g., lessening the patient's anxiety, providing comfort), important proximal outcomes of the clinician-patient interaction include patient understanding, trust, and agreement between the two parties, which can encourage both treatment adherence and better self-care skills [36].

Limited communication by healthcare providers to patients could be driven by a number of factors. As reported in the main study, providers rarely made a differential diagnosis for patients who tested negative, perhaps due to a shortage of diagnostic tools or limited patient examination arising from lack of clinical know-how, time or inclination, and thus were limited in the decisive information they were able to share [2]. Providers may also fail to appreciate the importance of communicating and interacting with patients, or lack adequate training in patient communication. Little data exists on patient expectations and communication desires during medical consultation in low-income settings [37]. The emphasis placed on communication skills during clinical training also lags far behind its importance in the patient-provider exchange during consultations [38]. Although patients almost always want as much information as possible, clinicians seem to underestimate patients’ desire for information [39].

We found inconsistencies in both the scope of information and terminology used to communicate test purpose and results, both across individual providers and across different providers and health facilities. The lack of consistency was unsurprising given the absence of clear guidance via a protocol for what should be communicated to patients, when and by whom. When test results were communicated, this was not consistently done so by the provider who conducted the test, but sometimes instead by the dispensing health worker alongside the provision of medication. While the involvement of a range of personnel can enhance quality of care—provided good clinical and laboratory practice are followed and systems are in place to support effective team work—disjointed clinical care does risk dehumanising the clinical interaction, and increases the likelihood of knowledge sharing gaps, inconsistencies or even contradictions in communication. This highlights the importance of addressing any gaps arising from the organisation of care, in addition to the improvement of inter-personal communication between providers and patients.

The fact that some patients who tested positive also reported feeling that they received insufficient information and explanation suggests that patient perceptions of the outcome may have more to do with a lack of healthcare provider communication than with the actual test result. In other words, acceptance of negative results may be less of an issue than feeling uninformed about the testing process, outcome and treatment plan more generally. The main study found that healthcare provider perceptions of patient demand for treatment, coupled with perceived patient rejection of test results, appeared to result in some anti-malarial prescription to patients who tested negative [2]. However this study found that RDT-negative patients generally did not request for specific drugs, suggesting that providers may both over-assume and over-report patient demand for drugs and that providers may misunderstand patients’ expectations and priority outcomes from the consultation. A review of the literature on patient-doctor communication asserts that communication is the main ingredient of medical care and that from the patient’s point of view, two needs have to be met when visiting the doctor: the ‘need to know and understand’ (to know what is the matter, where the pain comes from), and the ‘need to feel known and understood’ (to know the doctor accepts him and takes him seriously). Once the diagnosis and treatment plan is established, it is important that doctors efficiently impart this information to their patients [39]. Some RDT-negative patients in this study reported no benefit to testing, linking this closely to the absence of an alternative diagnosis or the receipt of treatment; effective communication on the test purpose, the test result and its meaning, and resultant clinical course of action may have alleviated this.

Interestingly, a recent study also conducted in mid-western Uganda showed that community members perceived quality of care as higher for VHTs, operating under the ICCM programme, than for health facility workers [40]. VHTs also have to manage RDT-negative patients and have fewer diagnostic tools and treatment options than health facility staff, though were equipped with visual job aids which supported explanations of the process of diagnosis and treatment of common childhood illnesses; comparable job aids were not always available at the facility level. It has been suggested that this higher perceived quality of care may be due to better communication on the part of VHTs with their already known subjects in their communities [40]. However, the communication patterns of VHTs’ encounters with patients has not been documented to date, making it difficult to foresee which aspects could be integrated into health workers’ practice in a health facility setting.

Finally, this study found that patients tended to be deferential to healthcare providers and rarely raised questions; providers were therefore seldom prompted by patients for more information and may have been unaware of patients’ desire for further explanation. This echoes findings from Ghana, where patient experiences with RDTs appeared to be embedded in existing hierarchical social relations between clinicians and patients, with patients perceiving limited ability to engage in the clinical process and to influence providers’ behaviour around the testing and treating process [20]. A study which explored adherence to antiretroviral therapy in South Africa further suggested that adherence was multifaceted, affected by a range of socio-cultural, economic, context and systemic issues, with analysis reinforcing the critical role of communication factors in achieving concordance between patient and pharmacist [41]. Patient participation in medical encounters clearly depends on a complex interplay of personal, physician, and contextual factors [42]. Ong suggests that three purposes of good communication between doctors and patients can be distinguished: creating a good inter-personal relationship, exchanging information and making treatment related decisions; all of these require the active participation of both patients and providers [39]. While critical for driving patient participation and thus quality of care, treatment adherence and improved patient outcomes, this study provides further evidence that effective consideration of the socio-cultural context is not adequately accounted for in the deployment of parasite-based diagnosis.

### Limitations

Assessment of, and ability to, interpret non-verbal communication could have been aided by video recordings of clinical observations though this was not done. However, the observed exchanges between healthcare providers and patients were frequently discussed between the two observers to support consistent interpretation and documentation of the patient-provider exchange. Although all attempts were made to ensure that healthcare providers continued ‘normal, everyday practice’, some observer-expectancy effect may have occurred, in that providers may have been more inclined to perform to a higher standard than usual. Finally, negative test results were not evenly distributed by age, limiting our ability to interpret any potential differences between adult and child patients, or between patients with positive and negative test results.

### Recommendations

The process through which RDTs are utilised to reach a decision about patients’ care should feature as more of a dialogue between healthcare provider and patient. This would allow providers the opportunity to understand better the patient’s condition, thus supporting the overall diagnostic process (through active and responsive—not just instructive—problem solving), as well as building patient trust and understanding of his/her own condition. A dialogical approach would also allow patients the opportunity to understand the clinical rationale behind care decisions, important if patients are to fully engage in the treatment plan, and which will ultimately drive perceptions of quality of care and future care seeking decisions [43, 44]. Improved healthcare provider communication skills are required. A stronger emphasis should be given to communication in clinical (including nursing) curricula, ad-hoc trainings, clinical mentoring and improved support supervision. Evaluations of supporting interventions introduced alongside RDTs to improve inter-personal communication skills and practice among health workers could support the prioritisation and design of appropriate strategies. The challenge faced by healthcare providers will be to accept change in an existing system of ‘opaque diagnosis’, embedded in a system of clinician authority and patient trust [20].

The absence of specific patient-provider communication standards should be addressed through the development of context-relevant communication protocols and job aids, drawing on case scenarios, expected to provide cues for clinicians to more systematically integrate information gathering and sharing into their interactions. Recent research in Uganda has identified patient-centredness as an aspiration for both health workers and community members for public health facilities [45], a useful foundation for the introduction of such initiatives. Protocols and communication guides should also provide clarification on what RDTs are able to detect.

Initiatives, which recognise and are suitably adapted to the local socio-cultural context, should be developed to generate demand for information among patients, who should be supported in understanding their health care rights and empowered to ask questions; their evaluation will also be critical to furthering understanding of local barriers and constraints. This study confirms that a shift in the patient-provider relationship from its former emphasis on paternalism, to a recognition of the importance of an informed and actively participating patient, is emerging as a growing need and desire. However, in low-resource settings where health workers are already stretched, it may not be realistic, nor desirable, to expect health workers to assume sole responsibility for actively engaging patients in the care process. Complementary interventions on the demand side should be explored to better inform patients and the general public on the care process, diagnostic tools and treatment options, with the aim of enabling patients to assume greater responsibility for the prevention, detection and treatment of health problems in a manner that supplements professional service. The strategic use of social and behaviour change communication, applying targeted messaging and tailored approaches, to promote a range of supportive preventive, care-seeking and disease management behaviours is vital for creating demand for testing and for building trust in results [46, 47], particularly when patients receive RDT-negative results and are unsure of what to do next. Communication interventions that support the adoption of diagnostic testing by both patients and providers is also a necessary step for improved treatment and surveillance of malaria, and increases the likelihood of a good return on investment for malaria programmes [48].

There are also implications for organisation of care. Clarifications on task management may be required, such as relating to who communicates test results (prescribing clinician or dispensing health worker). Where possible, physical spaces should be configured to allow for private, detailed patient history taking and assessment, and healthcare providers encouraged to respect patient confidentiality. Given RDTs were the first point-of-care diagnostic introduced at many lower level health facilities in Uganda and similar contexts, experience in their use may also have valuable implications for wider practice.

## Conclusions

Poor communication practices among healthcare providers around the testing process, as well as limited inter-personal exchange between providers and patients, had considerable bearing on patients’ perceptions of their proposed treatment. Patients have a right to health information and may be more likely to accept and adhere to treatment when they understand their diagnosis and treatment rationale in relation to their perceived health needs and expectations from their clinic visit. A more dialogical and patient-centred communication approach, guided by clear protocol, would help enable a more effective patient assessment, explanation of test capabilities and discussion on test outcomes in relation to proposed treatment, at the same time supporting the development of a more trusting relationship between provider and patient and allowing expectations to be clarified and questions to be asked. As well as improving overall quality of care, this will likely increase treatment adherence and further promote the demand for test-driven diagnoses. Socio-cultural contextual barriers are important and require detailed consideration in the planning of any training or other supporting interventions. Challenges are also created by the organisation of facility-level care, in particular task management, systems for information transfer and the physical organisation of space for patient care; these will also require attention under the overall aim of improving practice around the use of RDTs and related inter-personal communication.
